# Volkman's Contracture, Persistent Limb Ischaemia, and Amputation: A Complication of Brachial Artery Catheterisation for Haemodynamic Monitoring Using PiCCO

**DOI:** 10.1155/2013/474358

**Published:** 2013-09-26

**Authors:** Veejay Bagga, Marion Palmer, Ramesh Sadasivan, Govindan Raghuraman

**Affiliations:** Department of Intensive Care Medicine, Birmingham Heartlands Hospital, . Apartment 31, Queens View, 88 Park Grange Road, Sheffield, S2 3RY, Birmingham B9 5SS, UK

## Abstract

We report a case of a 64-year-old woman who was admitted to intensive care unit with multiorgan failure secondary to *Plasmodium falciparum* malaria. Haemodynamic monitoring using the transpulmonary thermodilution with pulse contour analysis system (PiCCO) was achieved via the left brachial artery. Two days later, a flexion deformity of the left hand was noted, and examination revealed left lower arm ischaemia. Removal of the catheter resulted in an immediate improvement of the contracture. However, distal pulses were still absent, and the arm remained ischaemic resulting in a below elbow amputation. This is the first documented case of a persistent limb ischaemia following the insertion of an arterial catheter for haemodynamic monitoring with PiCCO. We therefore highlight the need for regular assessment of limb perfusion after arterial catheterisation for haemodynamic monitoring with PiCCO. In addition, the brachial artery should be avoided, and femoral artery catheterisation is recommended.

## 1. Introduction

Advanced haemodynamic monitoring is used extensively in intensive care to help assist with cardiovascular management of critically ill patients. Traditionally, advanced haemodynamic management involved the use of pulmonary artery catheters (PAC); however, this method is becoming less popular as the benefits of using this technique remain controversial [1, 2]. In addition, its use has been associated with complications such as arrhythmias, infection, thrombotic complications [3], and, rarely, rupture of the pulmonary artery [4, 5]. 

Transpulmonary thermodilution monitoring using the PiCCO system (PULSION Medical System, Munich, Germany) is being used as a popular alternative to PAC. It's claimed benefits include minimal side effects, less invasiveness and is well validated against PAC. Data on the complications associated with this monitoring technique is limited; however, a recent prospective study which included over 500 patients in 14 different European intensive care units has shown that haemodynamic monitoring with PiCCO is safe, and serious complications are rare [6].

## 2. Case Report

A 64-year-old lady with recent history of traveling to Uganda was admitted to the hospital with *Plasmodium falciparum* malaria. Her comorbidities included hypertension and morbid obesity. The initial parasite count was 14%. Soon after her admission, her clinical condition deteriorated, and she developed multiple organ failure requiring ventilatory, renal supports, and advanced haemodynamic management. To help assist with cardiovascular and circulatory management, haemodynamic monitoring by transpulmonary thermodilution with arterial pulse contour analysis (PiCCO) was used. To achieve this, an arterial catheter was inserted into the left brachial artery under ultrasound guidance as multiple attempts at catheterising the femoral artery failed.

Distal limb perfusion was monitored; however, discolouration was difficult to recognise because of skin colour and the use of Henna on the hands and nails. Two days after the insertion of the arterial catheter, a Volkman's flexion contracture of the left hand was noted. On closer examination, the left lower arm was cold to touch, and capillary refill time was prolonged. In addition, no distal pulses were palpable, and hand held Doppler ultrasound could not record a trace. The arterial catheter was removed, and although immediate improvement of the contracture was observed, distal pulses still remained absent. After review by vascular surgeon, exploratory surgery of the brachial artery revealed local thrombus; hence, a thrombectomy was preformed and a vein patch was used to repair the artery. Also a limited fasciotomy of the forearm was performed but no underlying necrosis or swelling of the musculature was identified. Despite surgery, the left hand remained hypoperfused and ischaemic below the elbow. She required a below elbow amputation of the left arm. Her clinical condition further deteriorated as she developed bowel infarction, and surgery failed to improve her clinical condition. She eventually succumbed to her illness.

## 3. Discussion

This is the first documented case of persistent limb ischaemia and amputation following the use of the brachial artery for haemodynamic monitoring using PiCCO. 

Although ischaemia and pulse loss have been previously reported using the PiCCO system [6], these patients did not suffer permanent ischaemia as perfusion was restored when the catheter was removed. In addition, these patients underwent femoral and not brachial artery catheterisation. In studies that have used the brachial artery for haemodynamic monitoring, no complications have been documented [6, 7]. It is however important to note that data documenting the use of the brachial artery for haemodynamic monitoring is very limited. In the observational study by Belda et al., only 4 of the 514 patients reviewed had a PiCCO catheter inserted into the brachial artery. The majority of centres use the radial, femoral, or the axillary artery. The reason for this is unclear but may be because of the lack of collateral circulation of the brachial artery making it a high risk artery to catheterise [8]. Indeed, cannulation of the brachial artery has been used for other purposes, and median nerve injury along with ischaemia have been reported resulting in long-term disability [8, 9].

Review of the literature shows that the radial artery is the preferred choice for catheterisation for haemodynamic monitoring because of the reported low complication rates and ease of access [7]. Catheterisation of the radial artery is also preferred over the femoral artery because the latter is thought to be associated with increased infection risk due to its close proximity to the perianal area [10]. A number of studies; however, have shown no evidence of increased infection when using the femoral artery [8, 11, 12]. Furthermore, some authors suggest that haemodynamic monitoring is more accurate when using the femoral artery [7], thus indicating that the femoral artery may be a better option for catheterisation. 

In addition to the use of PiCCO, an important factor which may have contributed to this ischaemic episode is that the patient had severe malaria—a condition which is associated with hypercoagulation [13]. The combination of these two entities would enhance the risk of limb ischaemia; therefore coagulation status should be considered prior to inserting arterial lines for PiCCO. 

To conclude, we highlight the complications of arterial catheterisation for haemodynamic monitoring using PiCCO, particularly when using the brachial artery in patients with hypercoagulable states, for example, malaria. We suggest that distal circulation is monitored regularly after arterial catheterisation and that the femoral artery is used unless contraindicated.

## References

[1] Wiener RS, Welch HG (2007). Trends in the use of the pulmonary artery catheter in the United States, 1993–2004. *Journal of the American Medical Association*.

[2] Ospina-Tascón GA, Cordioli RL, Vincent J-L (2008). What type of monitoring has been shown to improve outcomes in acutely ill patients?. *Intensive Care Medicine*.

[3] Hadian M, Pinsky MR (2006). Evidence-based review of the use of the pulmonary artery catheter: impact data and complications. *Critical Care*.

[4] Kelly TF, Morris GC, Crawford ES (1981). Perforation of the pulmonary artery with Swan-Ganz catheters. Diagnosis and surgical management. *Annals of Surgery*.

[5] Della Rocca G, Costa MG (2004). Hemodynamic-volumetric monitoring. *Minerva Anestesiologica*.

[6] Belda FJ, Aguilar G, Teboul JL (2011). Complications related to less-invasive haemodynamic monitoring. *British Journal of Anaesthesia*.

[7] Scheer BV, Perel A, Pfeiffer UJ (2002). Clinical review: complications and risk factors of peripheral arterial catheters used for haemodynamic monitoring in anaesthesia and intensive care medicine. *Critical Care*.

[8] Cousins TR, O’Donnell JM (2004). Arterial cannulation: a critical review. *American Association of Nurse Anesthetists Journal*.

[9] Horlocker TT, Bishop AT (1995). Compartment syndrome of the forearm and hand after brachial artery cannulation. *Anesthesia and Analgesia*.

[10] Thomas F, Burke JP, Parker J (1983). The risk of infection related to radial vs femoral sites for arterial catheterizatin. *Critical Care Medicine*.

[11] Frezza EE, Mezghebe H (1998). Indications and complications of arterial catheter use in surgical or medical intensive care units: analysis of 4932 patients. *American Surgeon*.

[12] Traoré O, Liotier J, Souweine B (2005). Prospective study of arterial and central venous catheter colonization and of arterial- and central venous catheter-related bacteremia in intensive care units. *Critical Care Medicine*.

[13] Ghosh K, Shetty S (2008). Blood coagulation in falciparum malaria—a review. *Parasitology Research*.

